# RNA Interference of Human α-Synuclein in Mouse

**DOI:** 10.3389/fneur.2017.00013

**Published:** 2017-01-31

**Authors:** Young-Cho Kim, Adam Miller, Livia C. R. F. Lins, Sang-Woo Han, Megan S. Keiser, Ryan L. Boudreau, Beverly L. Davidson, Nandakumar S. Narayanan

**Affiliations:** ^1^Department of Neurology, University of Iowa Hospitals and Clinics, Iowa City, IA, USA; ^2^Aging Mind and Brain Initiative, University of Iowa Hospitals and Clinics, Iowa City, IA, USA; ^3^Department of Physiology, Federal University of Sergipe, São Cristóvão, Brazil; ^4^Children’s Hospital of Philadelphia, Philadelphia, PA, USA; ^5^Department of Internal Medicine, University of Iowa Hospitals and Clinics, Iowa City, IA, USA

**Keywords:** RNA interference, α-synuclein, Parkinson’s disease, siRNA, neurodegeneration

## Abstract

α-Synuclein is postulated to play a key role in the pathogenesis of Parkinson’s disease (PD). Aggregates of α-synuclein contribute to neurodegeneration and cell death in humans and in mouse models of PD. Here, we use virally mediated RNA interference to knockdown human α-synuclein in mice. We used an siRNA design algorithm to identify eight siRNA sequences with minimal off-targeting potential. One RNA-interference sequence (miSyn4) showed maximal protein knockdown potential *in vitro*. We then designed AAV vectors expressing miSyn4 and injected them into the mouse substantia nigra. miSyn4 was robustly expressed and did not detectably change dopamine neurons, glial proliferation, or mouse behavior. We then injected AAV2-miSyn4 into Thy1-hSNCA mice over expressing α-synuclein and found decreased human α-synuclein (hSNCA) in both midbrain and cortex. In separate mice, co-injection of AAV2-hSNCA and AAV2-miSyn4 demonstrated decreased hSNCA expression and rescue of hSNCA-mediated behavioral deficits. These data suggest that virally mediated RNA interference can knockdown hSNCA *in vivo*, which could be helpful for future therapies targeting human α-synuclein.

## Introduction

α-Synuclein is an unstructured soluble protein involved in presynaptic processing of neurotransmitters, mitochondrial function, and proteasome processing (1, 2). In Parkinson’s disease (PD), α-synuclein aggregates in Lewy bodies, which contributes to cellular dysfunction (3). Mutations and polymorphisms of the human synuclein (hSNCA) gene have been identified in the brain tissues of patients with PD (4). Humans with α-synuclein gene duplications have a dramatically increased risk of PD (5, 6). Mice engineered to overexpress mutant human α-synuclein (A53T) in CNS neurons have profound neurodegeneration with characteristic histopathologic features of PD, marked behavioral dysfunction, and motor impairments (7). Mouse models with virally overexpressed α-synuclein in the midbrain recapitulate behavioral dysfunction and motor impairments (8, 9). These data implicate α-synuclein as a contributing protein in PD.

Although α-synuclein is involved in a variety of crucial physiological functions (10), α-synuclein knockout mice have relatively few neuroanatomical and motor deficits (11, 12) and are somewhat resistant to neurotoxins targeting dopamine neurons (13, 14). Thus reduction of α-synuclein has the potential to disrupt the chain of events causing protein aggregation and cell death in PD. In addition, α-synuclein in the cerebral cortex may contribute to Lewy-body dementia (DLB) and dementia in PD (15).

These data suggest that decreasing α-synuclein expression might be a viable neuroprotective strategy. Various strategies have been used to knockdown α-synuclein (16–20). Early studies using virally mediated RNAi targeting rat α-synuclein resulted in dopaminergic neuron toxicity (17, 18). A subsequent study subsequently used a microRNA-based strategy which yielded some neuroprotection but also inflammation and reduced expression of tyrosine hydroxylase, the rate-limiting enzyme for dopamine synthesis (21). Knocking down rodent α-synuclein can be protective in toxin-based animal models (20). In the present study, we harnessed bioinformatic algorithms to design small, interfering RNAs (siRNAs, 22–25). This technique has the potential to facilitate the safe and effective knockdown of toxic proteins in brain (26, 27). Here, we used this approach to design an RNAi targeting human α-synuclein and tested this approach *in vitro* and *in vivo* in mice.

## Results

### *In Vitro* Silencing of hSNCA

**Figure 1:**
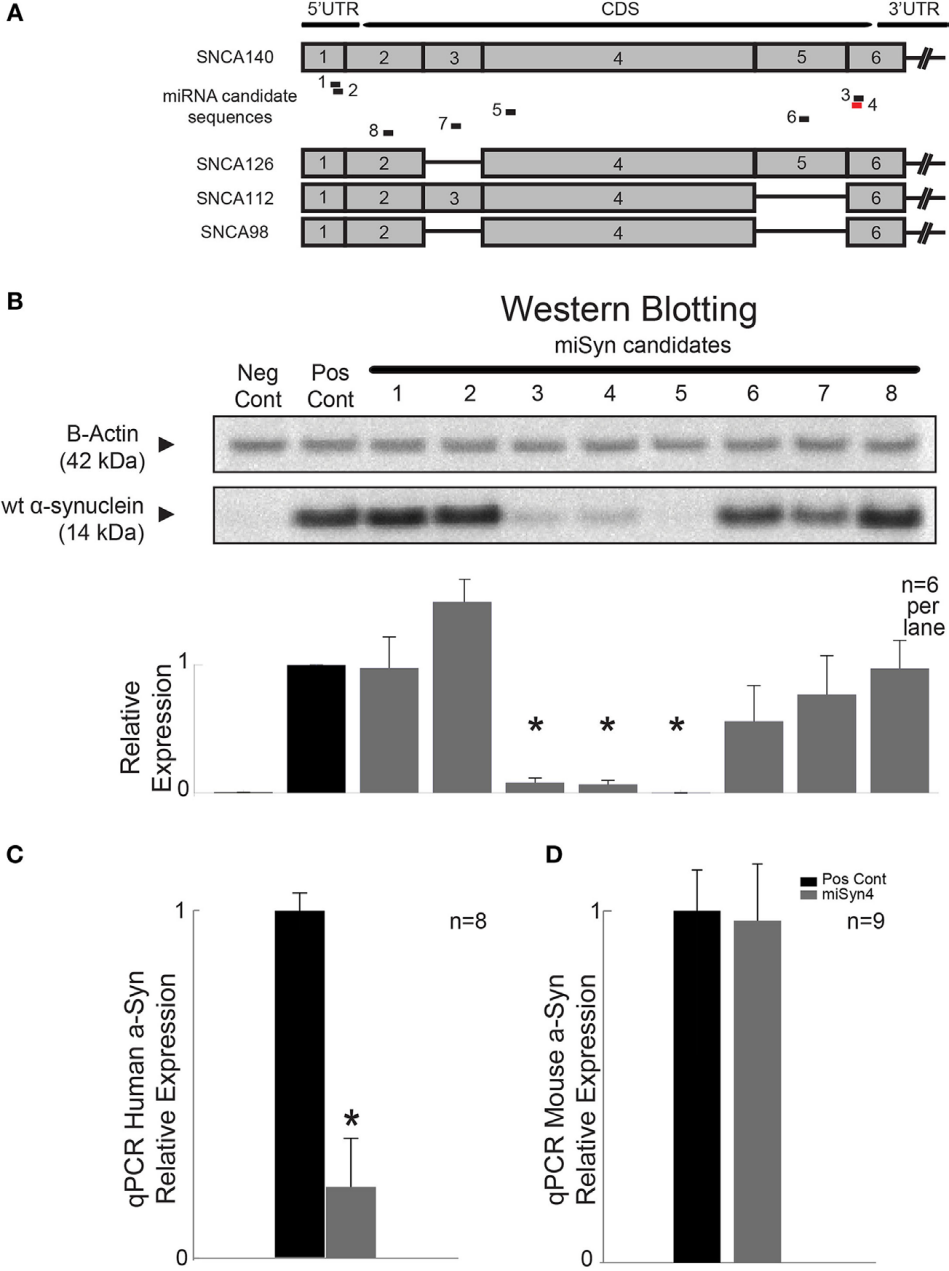
**RNA interference of α-synuclein**. **(A)** Design of RNAi molecules based on limited potential off-targeting revealed eight possible sequences, labeled miSyn1–8 (see Tables 1 and 2). miSyn1, 2, 3, 4, and 8 targeted to hSNCA140 and splice variants hSNCA126, 112, and 98. **(B)** In HEK293 cells, western blotting revealed marked knockdown of hSNCA for miSyn3–5; *n* = 6 samples per lane. Scrambled RNAi used a positive control. **(C)** miSyn4 knocked down hSNCA *in vivo* by qPCR; *n* = 8 samples. **(D)** miSyn4 did not affect mouse SNCA by qPCR; *n* = 9 samples. *p < 0.05 by *t*-test.

The goal of the present study was to test if virally mediated RNAi decreased expression of human α-synuclein in mouse models. We used siRNA sequence probability-of-off-targeting reduction (siSPOTR) tools to identify siRNA sequences with minimal off-targeting potential and identified eight candidate sequences, labeled miSyn1–8 (Figure 1A; Table 1). We tested the efficacy of these eight sequences by cotransfecting hSNCA and miSyn1–8 into HEK293 cells and assessed protein expression at 48 h by western blot. The cotransfection of miSyn3–5 showed reduced hSNCA protein expression relative to controls (miSyn3; 8 ± 4% of control, *t* = 24, *p* < 0.02; miSyn4 7 ± 3% of control, *t* = 29, *p* < 0.001; miSyn5 0 ± 0% of control, *t* = 745, *p* = 0; Figure 1B; *n* = 6 samples for miSyn1–8). Other clones were not effective. Based on this *in vitro* data and the fact that miSyn4 showed limited silencing against rodent synuclein (Table 2), we selected miSyn4 for further characterization.

**Table 1:** **RNA-interference sequences**.

Clone	Sequence
miSyn1	CTCGAGTGAGCGATGGGAGTGGCCATTCGATGAACTGTAAAGCCACAGATGGGTTCGTCGAATGGCCACTCCCAGCGCCTACTAG
miSyn2	CTCGAGTGAGCGAGGGAGTGGCCATTCGACGATACTGTAAAGCCACAGATGGGTGTCGTCGAATGGCCACTCCCACGCCTACTAG
miSyn3	CTCGAGTGAGCGTGGGTATCAAGACTACGAATTACTGTAAAGCCACAGATGGGTGGTTCGTAGTCTTGATACCCTCGCCTACTAG
miSyn4	CTCGAGTGAGCGTAGGGTATCAAGACTACGAATACTGTAAAGCCACAGATGGGTGTTCGTAGTCTTGATACCCTTCGCCTACTAG
miSyn5	TCTAGTAGGCGGACCAAAGAGCAAGCGACAAATCCCATCTGTGGCTTTACAGATTTGTCACTTGCTCTTTGGTTCGCTCACTCGAG
miSyn6	TCTAGTAGGCGATGCCTGTGGATCCCGACAATACCCATCTGTGGCTTTACAGTATTGTCAGGATCCACAGGCAGCGCTCACTCGAG
miSyn7	TCTAGTAGGCGAACCAAGGAGGGAGTGGCGCAACCCATCTGTGGCTTTACAGTTACACCACTCCCTCCTTGGTGCGCTCACTCGAG
miSyn8	TCTAGTAGGCGAAGGCCAAGGAGGGAGTCGTAACCCATCTGTGGCTTTACAGTCACAACTCCCTCCTTGGCCTGCGCTCACTCGAG

*A specificity-focused siRNA design algorithm identified microRNAs with minimal off-targeting potential labeled miSyn1–8*.

**Table 2:** **miSyn target sequence characteristics**.

miSNCA candidates	Silencing?	Target sequence conserved?								
	α-Synuclein	SNCA 126	SNCA 112	SNCA 98	Mouse/rat	β-Synuclein	γ-Synuclein	A30P	E46K	A53T
Optimal	Yes	Yes	Yes	Yes	No/no	No	No	Yes	Yes	Yes
miSNCA1	No	Yes	Yes	Yes	No/no	No	No	Yes	Yes	Yes
miSNCA2	No	Yes	Yes	Yes	No/no	No	No	Yes	Yes	Yes
miSNCA3	Yes	Yes	Yes	Yes	No/no	No	No	Yes	Yes	Yes
miSNCA4	Yes	Yes	Yes	Yes	No/no	No	No	Yes	Yes	Yes
miSNCA5	Yes	Yes	Yes	Yes	Yes/yes	No	No	Yes	Yes	Yes
miSNCA6	No	Yes	No	No	Yes/no	No	No	Yes	Yes	Yes
miSNCA7	Marginal	No	Yes	No	Yes/yes	No	No	Yes	No	No
miSNCA8	No	Yes	Yes	Yes	Yes/yes	Yes	Yes	Yes	Yes	Yes

*Summary of each clone’s target sequence, efficacy of silencing, coverage of major splice variants of human α-synuclein, and coverage of dominant pathogenic mutations*.

Next, we tested the efficacy of miSyn4 by qPCR. We cotransfected hSNCA and miSyn4 as well as a scrambled control sequence in HEK 293 cells (positive control in Figures 1B–D) and measured hSNCA levels at 48 h *via* qPCR. We found that there was significantly less hSNCA in cells transfected with miSyn4 compared to positive controls (*t* = 22, *p* < 0.03; *n* = 8; Figure 1C). We also transfected a separate group of HEK293 cells with mouse α-synuclein and measured levels of mouse α-synuclein at 48 h *via* qPCR. We found that miSyn4 did not influence the levels of mouse SNCA *in vitro* as measured by qPCR (*n* = 9; Figure 1D). These data demonstrate the miSyn4 can decrease expression of hSNCA *in vitro*.

### AAV2-miSyn4 Expression *In Vivo*

**Figure 2:**
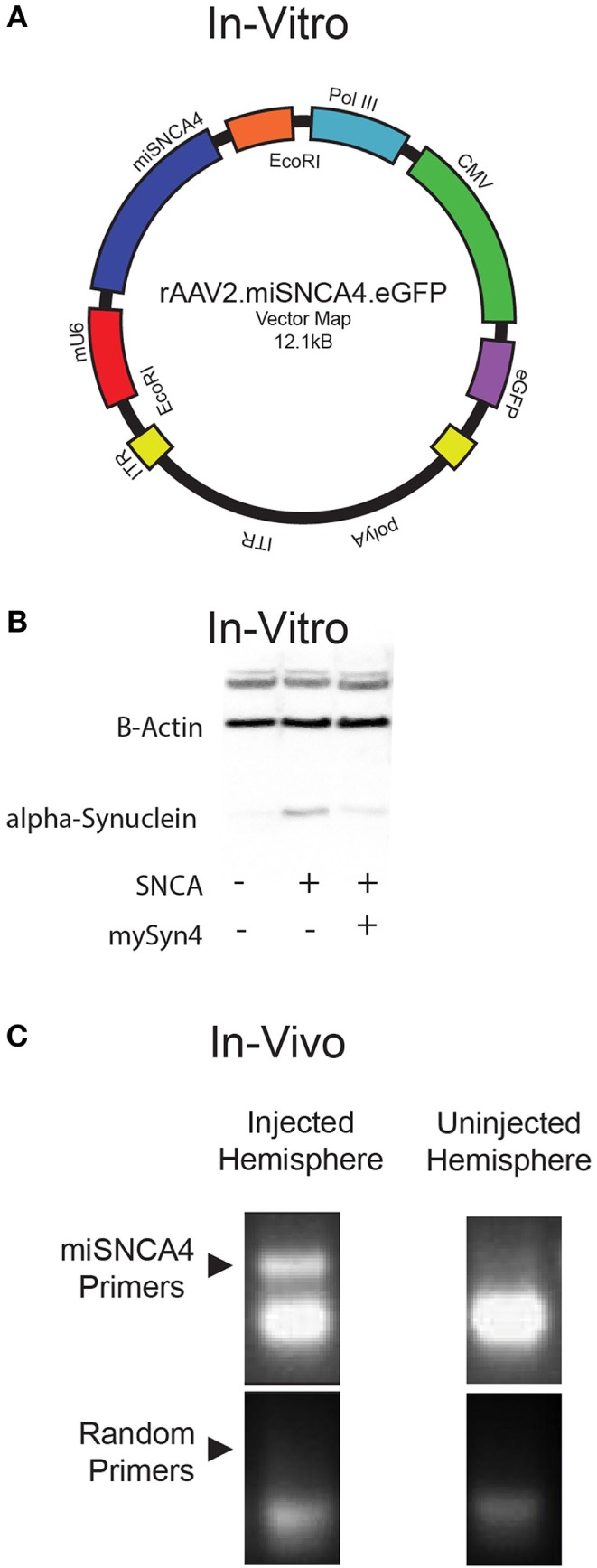
**Virally mediated knockdown of α-synuclein**. **(A)** Plasmid map of α-synuclein for AAV2-miSyn4. **(B)** Example of hSNCA in HEK293 cells transfected with AAV2.miSyn4 and hSNCA (right most lane); B-actin is present in all three lanes. **(C)** Example of semi-qPCR for miSyn4 from injected and uninjected cerebral hemispheres injected with AAV2-miSyn4.

**Figure 3:**
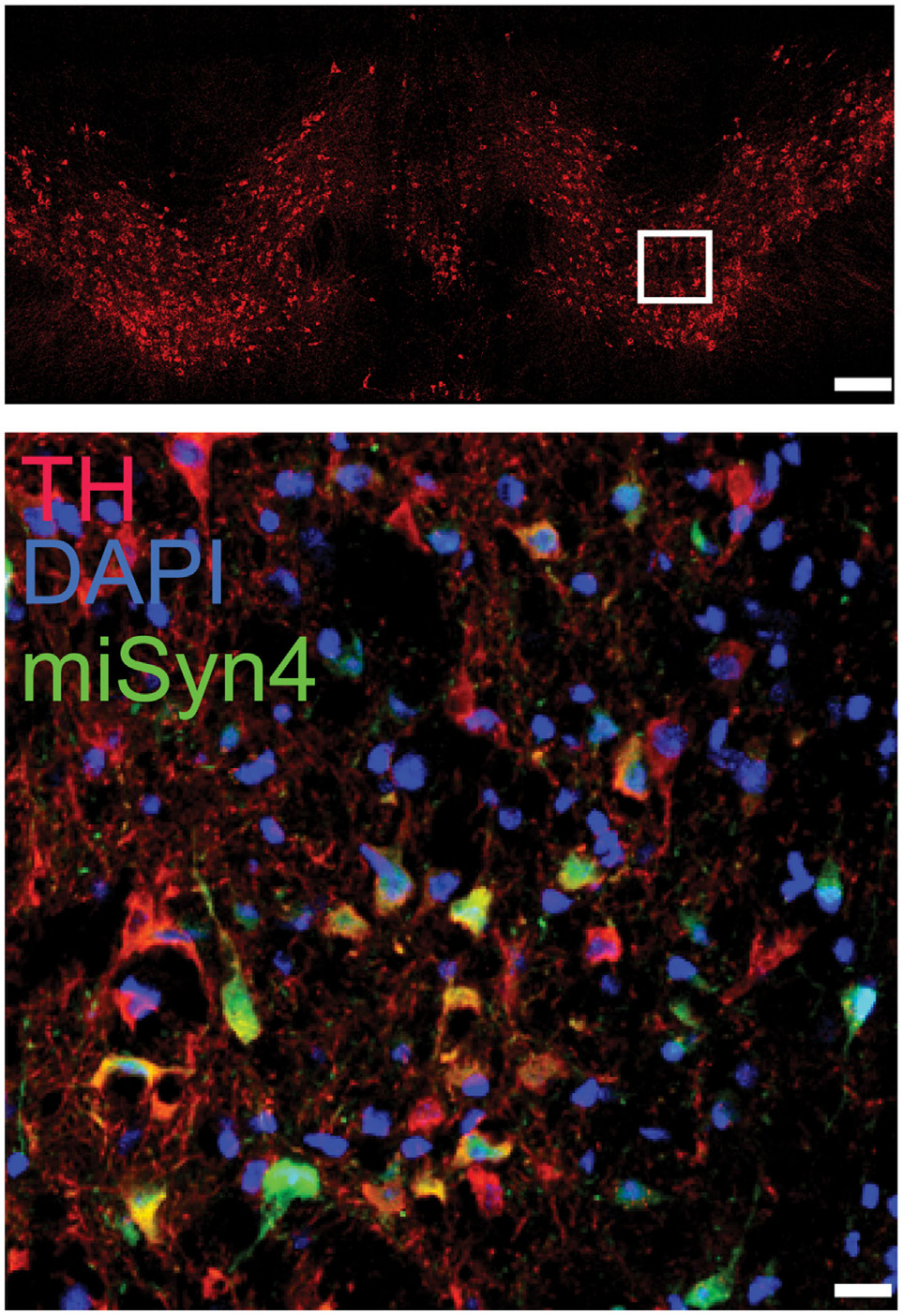
**Example immunohistochemistry from a mouse injected in the substantia nigra (top panel; scale bar—100 µm) with AAV-miSyn (green; bottom panel; scale bar—10 μm)**. Tyrosine hydroxylase in red.

Next, we wanted to test miSyn4 expression *in vivo*. We cloned miSyn4 into a vector co-expressing eGFP to facilitate visualization of viral transduction (22) (Figures 2A–C). AAVs have been used extensively to express RNAi molecules *in vivo* (28). We tested the efficacy of the miSyn4 expression system by injecting AAV2-miSyn4 into the substantia nigra of wild-type mice and examining expression of miSyn4. Semiquantitative PCR found miSyn4 expression of mice only in the injected side (Figure 2C). miSyn4 was expressed in both TH+ and TH− cells (6 ± 2 TH+ eGFP+ cells/high power field vs 4 ± 1 DAPI+ eGFP+ cells/high power field; *n* = 5 mice; Figure 3).

**Figure 4:**
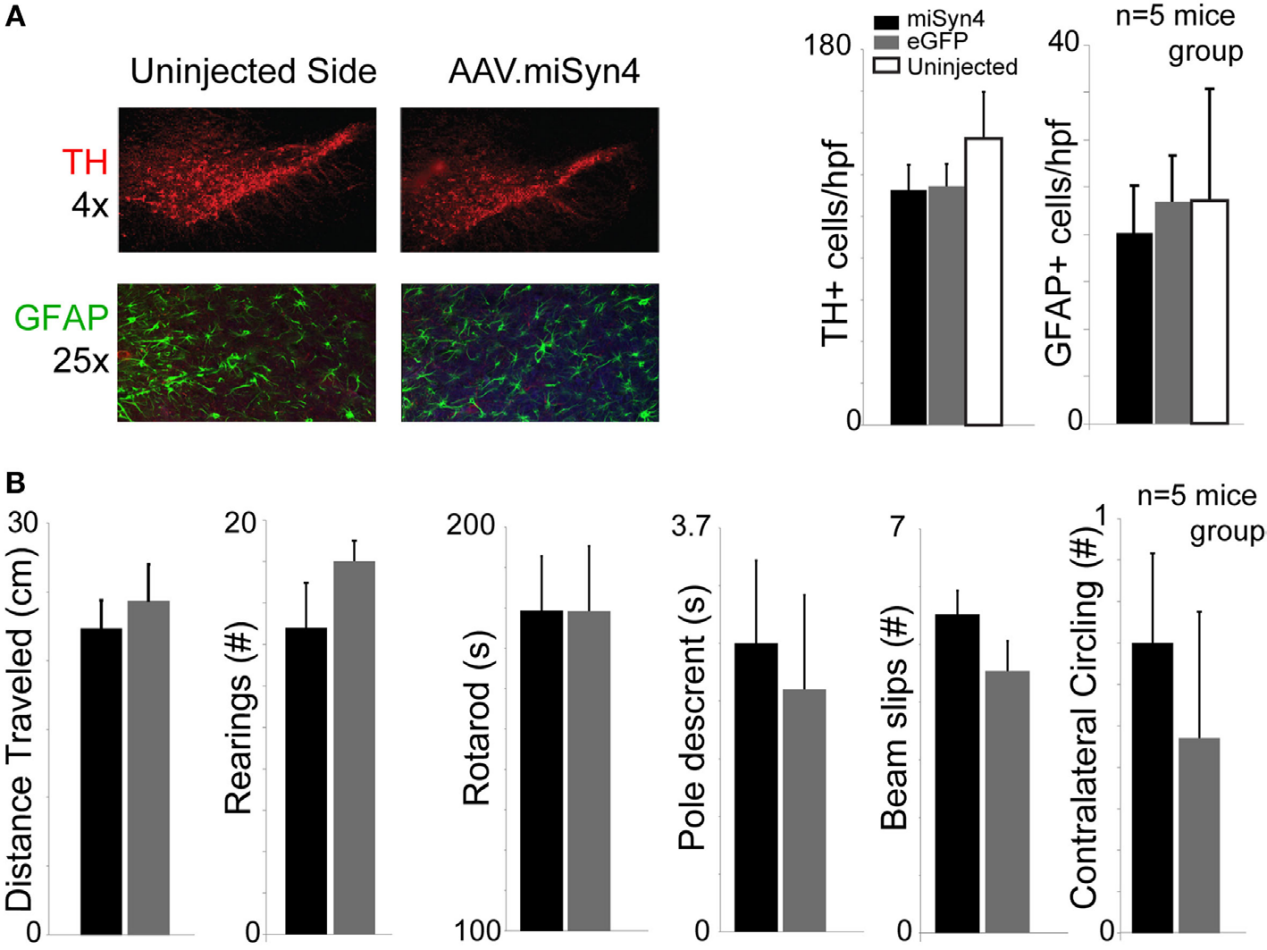
**AAV2-miSyn4 is tolerable to mice after 90 days**. **(A)** Mice injected with AAV2-miSyn4 had similar numbers of TH+ and GFAP+ cells by immunohistochemistry compared to uninjected sides or AAV-eGFP mice. Cell counts per high power field. **(B)** No behavioral differences were observed between mice injected with AAV-eGFP or AAV2-miSyn4 in open-field testing, number of rearings, rotarod latency, balance beam pole-traverse, balance beam slips, or contralateral circling. *n* = 5 mice per group; black bars—AAV-miSyn2; gray bars—AAV-eGFP, white bars—uninjected side.

Previous studies have reported decreased TH+ expression with α-synuclein RNAi (16). To examine if miSyn4 produced affected the number of TH+ cells, we counted the number to TH+ cells and GFAP+ cells in the midbrain of mice 90 days after mice were unilaterally injected with AAV2-eGFP or AAV2-miSyn4 in the substantia nigra. We found that AAV2-miSyn4 did not change TH+ cell or GFAP+ cell counts by fluorescent immunohistochemistry (Figure 4A; *n* = 5 mice). Finally, AAV2-miSyn4 expression for 90 days did not induce detectable behavioral changes as measured by open-field activity, number of rearings in the cylinder test, rotarod latency, the time to traverse a balance beam, beam slips, or contralateral circling (Figure 4B; *n* = 5 mice). Mice with dysfunctional dopamine neurons are impaired in these tasks (29). These data indicate thatAAV2-miSyn4 can be tolerable in wild-type mice.

### AAV2-miSyn4 Attenuates hSNCA Overexpression *In Vivo*

**Figure 5:**
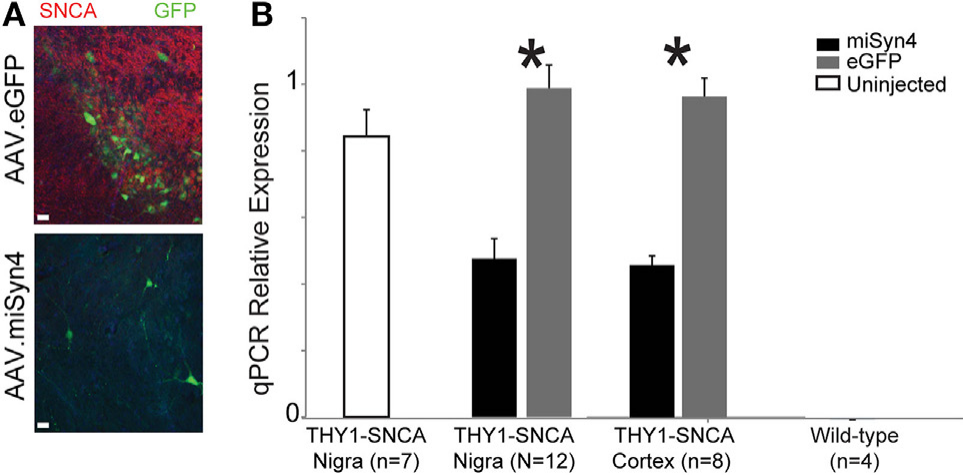
**AAV2-miSyn4 decreases α-synuclein in *Thy1-SNCA* mice after 90 days**. **(A)** Injection of AAV2-miSyn4 into the substantia nigra decreased hSNCA immunoreactivity using a human-specific synuclein antibody, **(B)** qPCR of α-synuclein from Thy1-SNCA mice revealed significantly less α-synuclein in mice injected with AAV2-miSyn4 in both the substantia nigra and the cerebral cortex. Sample size as shown; *p < 0.05 by *t*-test.

Thus far, we have demonstrated the miSyn4 decreases α-synuclein *in vitro* and can be expressed in mice without overt neuroanatomical or behavioral changes. We turned to two animal models of α-synuclein overexpression to test if AAV2-miSyn4 can effectively reduce hSNCA expression *in vivo*. First, we investigated the Thy1-SNCA mouse (30, 31), which overexpresses wild-type hSNCA throughout the nervous system. This mouse was selected as a proof-of-principle model for high levels in *in vivo* expression; we selected 2-month-old mice because we wanted to study α-synuclein expression prior to the onset of marked neurodegeneration (32). We injected AAV2-miSyn4 or AAV2-eGFP into the substantia nigra of 2-month-old Thy1-SNCA mice and examined SNCA expression by qPCR (Figure 5A). AAV2-miSyn4 significantly decreased hSNCA expression in the substantia nigra (47 ± 6 vs 98 ± 7% relative to eGFP injected controls; *t* = 5.6; *p* < 0.002; *n* = 12 mice) and in the medial frontal cortex (45 ± 3 vs 96 ± 6%; *t* = 7.4, *p* < 0.0003; *n* = 8 mice; Figure 5B). Notably, because AAV2-miSyn4 is only effective close to the injection site and α-synuclein in Thy1-SNCA mice is widely overexpressed throughout the nervous system (32), we did not study behavior in these animals.

We turned to a second model of α-synuclein overexpression using viral hSNCA overexpression using AAV2. Viral overexpression of hSNCA in the substantia nigra can model behavioral aspects of PD (9). To test whether behavioral deficits induced by hSNCA overexpression in substantia nigra can be alleviated by AAV2-miSyn4, four groups of mice were unilaterally injected into the substantia nigra with (1) AAV2-eGFP (vector controlling for miSyn4), (2) AAV2-mCherry (vector controlling for hSNCA), (3) AAV2-hSNCA with AAV2-eGFP, or (4) AAV2-hSNCA with AAV2-miSyn4. Ninety days after virus injection, a series of behavioral tests were performed and hSNCA levels were examined by western blot.

**Figure 6:**
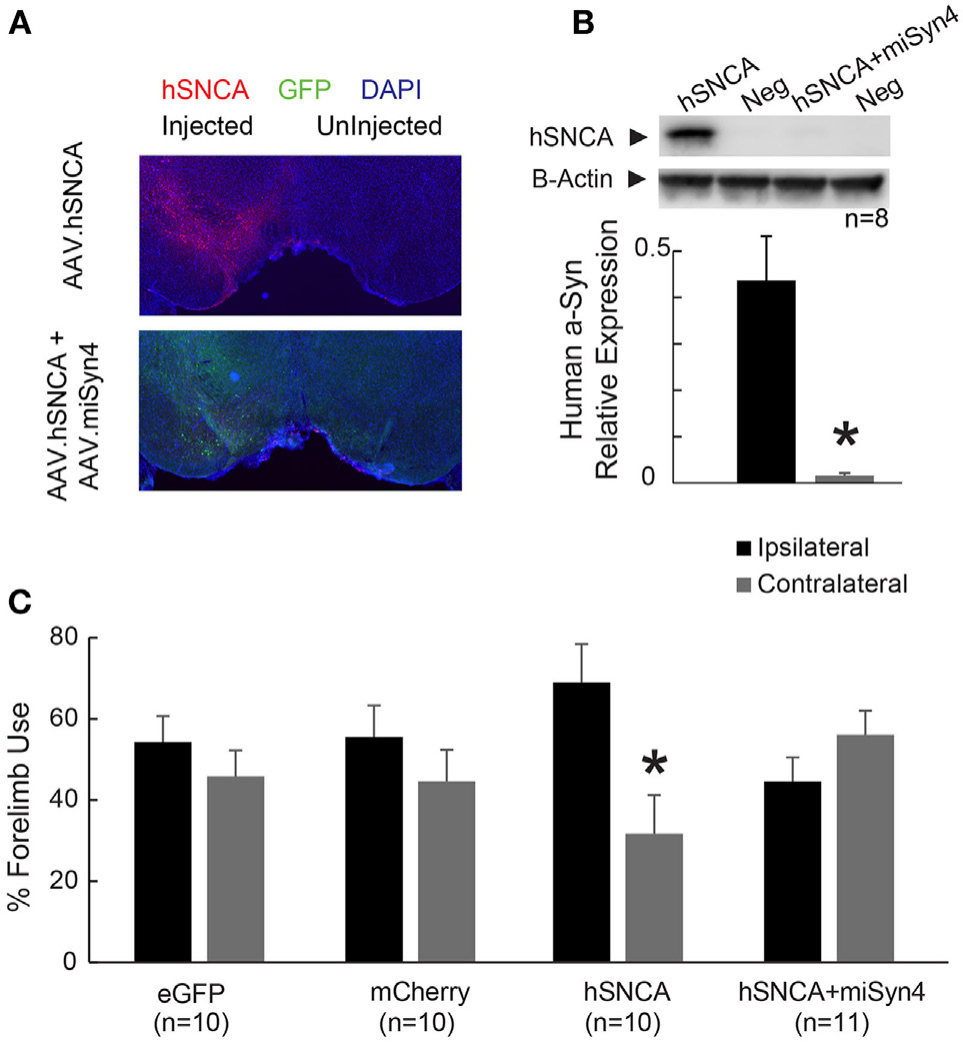
**AAV2-miSyn4 decreases human α-synuclein expression by AAV2-hSNCA**. **(A)** Injection of AAV2-hSNCA into substantia nigra induced hSNCA immunoreactivity, and co-injection of AAV2-hSNCA and AAV2-miSyn4 decreased hSNCA immunoreactivity. **(B)** Western blot of hSNCA from mice with co-injection of AAV2-hSNCA and AAV2-miSyn4 revealed significantly less hSNCA in mice injected with AAV2-miSyn4 in the substantia nigra. **(C)** Silencing of hSNCA expression in the substantia nigra by AAV2-miSyn4 improved motor deficits in AAV2-hSNCA injected mice, as measured by forelimb preference. *p < 0.05 *via* general linear model with Tukey *post hoc* test.

AAV2-hSNCA was expressed in the substantia nigra, and AAV2-miSyn4 reduced hSNCA expression (Figure 6A). Western blots indicated that overexpressed hSNCA was reduced in mice injected with AAV2-hSNCA and AAV2-miSyn4 compared to mice injected with AAV2-hSNCA alone (Figure 6B; 0.43 ± 0.096 vs 0.015 ± 0.0006; ~4% expression; *t* = 4.68, *p* < 0.0001; *n* = 8).

We next used the cylinder test to evaluate forelimb use asymmetry in these mice 90 days after unilateral injection of AAV2-eGFP, AAV2-mCherry, AAV2-hSNCA with AAV2-eGFP, or AAV2-hSNCA with AAV2-miSyn4 (9). While mice with unilateral injection of AAV2-hSNCA showed a preference for ipsilateral forelimb use and a deficit in contralateral forelimb use, mice with unilateral injection of AAV2-hSNCA + AAV2-miSyn4 showed no preference or deficit on forelimb use (Figure 6C; 44.3 ± 6 vs 55.7 ± 6%, two-way ANOVA with Tukey *post hoc* test, *p* = 0.017; *n* = 10). AAV2-hSNCA did not reliably impair rotarod and open-field circling; hence, we did not focus on these assays. Taken together, these data indicate that virally expressed miSyn4 can attenuate some effects of hSNCA overexpression *in vivo* in two animal models.

## Discussion

In the present study, we designed an RNAi-based approach to decrease hSNCA in mouse models. We used bioinformatic algorithms to engineer siRNA targeting hSNCA and used AAV2 to express this molecule *in vivo*. We found that it could be tolerated in mice and could effectively reduce expression of hSNCA in two mouse models that overexpress hSNCA. α-Synuclein appears to be involved in endocytosis of vesicles (33). Accelerated fibril formation appears to contribute to Lewy-body formation in PD (34, 35), and these fibrils could be transmissible through prion-like mechanisms (36–38). α-Synuclein has been implicated as a key molecule in PD and thus is an attractive target for novel therapeutic interventions (4, 39). Although there have been several attempts to decrease α-synuclein through a variety of methods, ours is the first to demonstrate *in vivo* effectiveness against human α-synuclein.

Prefacing this work is extensive data on mice with α-synuclein knocked out; in these mice, there is no neurodegeneration and mild decreases in dopamine content (11). However, α-synuclein knockout mice can be resistant to neurotoxins such as MPTP and 6-OHDA (13, 40). These data indicate that α-synuclein could be an attractive target for neuroprotective therapies. RNAi approaches targeting α-synuclein have had mixed results. Knocking down α-synuclein in primates by direct infusion of siRNA also resulted in decreased α-synuclein expression without neurotoxicity (19). However, AAV-mediated shRNA against rat α-synuclein reported marked reduction in TH+ neurons in the midbrain (16). A subsequent study targeting rat α-synuclein found no toxicity and reported neuroprotection in a rotenone model of PD (20). RNAi approaches targeting human α-synuclein in rats have also resulted in some toxicity and inflammation that may have been dependent on the level of shRNA expression (17, 18, 21). Here, we use bioinformatic approaches to minimize off-targeting and knockdown human α-synuclein in mice. Our results are novel on several fronts. First, whereas prior work was in rats or primates, our work is in mice, enabling us to provide new data in transgenic mice overexpressing α-synuclein (32). Second, whereas some prior work in rats had toxicity or inflammation (16, 18, 21), our approach, which harnessed an informatics approach, did not find evidence of toxicity or motor deficits. Third, we provide novel evidence that in two mouse models we can decrease α-synuclein overexpression as well as some aspects of behavior.

We decreased α-synuclein in a restricted cell-population around our injection site in the midbrain. We intentionally did not knockdown mouse synuclein (13, 14). This limits our ability to study if AAV2-miSyn4 protects against neurotoxins in mouse models (41). α-Synuclein is expressed in the cortex, as well as the midbrain (42), and may contribute to cognitive impairments in PD (43–45) and DLB (15, 46). Our data indicate that virally mediated RNAi for hSNCA was effective in the cortex as well as the midbrain. These data could be helpful in developing neuroprotective strategies for human synucleinopathies such as PD and DLB.

We used a virus with AAV2/5, which we have used in the past to robustly and reliably express genes in the rodent brain (26, 43, 47, 48). While there are many serotypes of AAV that can be effective in the brain (49), AAV2 is currently in clinical trials for PD (NCT# NCT0162158). Future studies will compare AAV serotypes to achieve maximal expression with minimum toxicity.

Our study has several limitations. First, we use two models of α-synuclein overexpression in mice: the Thy1-SNCA mouse and viral hSNCA overexpression (32, 50). These models are very different from clinical PD, which can be quite complex and involve many cellular and circuit processes. The Thy1-SNCA mouse expresses α-synuclein under the Thy1 promoter at very high levels. These may include non-dopaminergic neurons, neurons outside the nigra, and may encompass non-neuronal tissues such as glia and endothelial cells. Unraveling these interactions will likely involve systematically manipulating α-synuclein in each of these cell types, but could have particularly relevance to PD as well as other synucleinopathies, such as multiple-systems atrophy and dementia with Lewy bodies (4, 15). Although few studies have explored viral SNCA overexpression in mice (9), we find behavioral recovery with forepaw preference in the viral hSNCA model, constraining our ability to find evidence of neuroprotection. Our behavioral effects are limited to this assay; mostly because hSNCA mice did not have reliable motor deficits in other tests of motor function in our hands. One challenge is that mouse models of α-synuclein overexpression in mice do not consistently involve robust motor impairments at a young age; indeed, often the animals must be aged or involve multiple mutations, or broad synuclein overexpression in many tissues and brain areas (15, 30, 32). Future work could examine motor deficits in these animals as they age, as well as examine mice with synuclein mutations or protofibril deposition (7, 37). We also did not look directly at cell death in our hSNCA overexpression experiments. Human SNCA overexpression can lead to marked cell death in rats, although in mice this appears to be somewhat less (9, 51). Furthermore, synuclein can affect complex aspects of dopamine neuron biology, including vesicles and dopamine release (52). Notably, we determine how levels of α-synuclein measured in hSNCA overexpressing animals are affected by cell death. Notably, these experiments are all done in mice and do not indicate whether this approach would be well received should the virus be injected in to humans. Future work will be required to establish whether RNAi is a safe and effective strategy in humans *in vivo*.

## Materials and Methods

### microRNAs

microRNAs were designed using the siSPOTR tool (23) to maximize interference potency and minimize potential off-target sequences (24). Eight potential sequences (miSyn1–8) targeting exons of the human α-synuclein gene (SNCA; OMIM). These sequences had low off-targeting potential (Table 1). Artificial miRNA expression cassettes were cloned into Tb:mU6 expression plasmids, and recombinant AAV serotype 2/5 vectors (AAV2-miSyn and AAV2-miSCA1) were generated by the University of Iowa Vector Core facility as previously described (53). AAV vectors were resuspended in buffer, and titers were determined by qPCR. Control vectors were identical plasmids without miSyn expression cassettes. Viruses were stored in a salt solution and dialyzed prior to injection (54).

### *In Vitro* Analysis

HEK293 cells were obtained from the Gene Vector Core at the University of Iowa. Cells were plated with equal density (400,000 cells/ml) in 24-well plates. The next day, cells were cotransfected with PCEP4-SNCA + miSyn candidates using lipofectamine 2000 transfection reagent. Forty-eight hours later, cells were lysed for analysis of protein expression by western blot and RNA expression by qPCR. Control cells expressed PCEP4-SNCA + a U6 scrambled snRNA control (to balance RNA content) or were untreated HEK293 cells.

### Animals

Sixteen wild-type ~3-month-old C57/B6 male mice were used to assess the expression and tolerability of miSyn. Twenty-one additional transgenic ~3-month-old Thy1-SNCA mice were used to test miSyn in transgenic models of α-synuclein (32, 55). These mice express α-synuclein in a variety of tissues under the Thy1 promoter and have severe behavioral deficits due to ubiquitous α-synuclein overexpression (31). Forty 3-month-old wild-type mice were injected with AAV2-hSNCA, AAV2-miSyn4, or control viruses (an identical vector to AAV-miSyn4 without miRNAi, with GFP driven under the CMV promoter) to test if miSyn4 could rescue behavioral deficits caused by AAV2-hSNCA overexpression. All animals were housed with littermates using a 12-h light/dark cycle. This study was carried out in strict accordance with the recommendations in the Guide for the Care and Use of Laboratory Animals of the National Institutes of Health. The protocol was approved by the Institutional Animal Care and Use Committee at the University of Iowa (protocol # 4071105). All surgery was performed under ketamine and xylazine anesthesia, and all efforts were made to minimize suffering.

### AAV Injections

Mice were anesthetized with ketamine (100 mg/kg) and xylazine (10 mg/kg) and placed in a stereotaxic frame. The scalp was retracted, bregma and lambda were leveled, and a small craniotomy was drilled. Coordinates for the substantia nigra were AP: −3.3 mm, ML: −1.2 mm, and DV: −4.5 mm and for cortical injections were AP: +1.5 mm, ML: −1.0 mm, and DV: −1.5 mm. To locally overexpress wild-type hSNCA in the mouse substantia nigra, an hSNCA (BC013293.2) vector driven by CAG promoters was designed with recombinant AAV2/6 vectors (serotype 2 genome/serotype 6 capsid; this vector was distinct from AAV2/5 made by the University of Iowa vector core for miSyn above) (9) CAG-hSNCA-WPRE and CAG-mCherry-WPRE vectors were produced and purified in Vector BioLabs (Philadelphia, PA, USA). AAV titers were ~10^13^ GC/ml by qPCR.

### Histology

Mice were anesthetized with a ketamine/xylazine mix and transcardially perfused with 20 ml of 0.9% cold saline. For histological analyses, mice were decapitated, and brains were removed and postfixed overnight in 4% paraformaldehyde. Brains were stored in a 30% sucrose solution at 4°C, sectioned on a freezing microtome at 40 µm thickness and stored at −20°C in a cryoprotectant solution. For RNA sample preparation, the brain was triturated in 1 ml of TRIzol (Life Technologies, Grand Island, NY, USA), flash frozen in liquid nitrogen, and stored at −80°C until used. RNA was isolated from tissue expressing eGFP under fluorescence microscopy using 1 ml of TRIzol. RNA quantity and quality was measured using a NanoDrop^®^ ND-1000 (Nanodrop, Wilmington, DE, USA).

### Immunohistochemical Analyses

Free-floating coronal sections (40-µm thick) were washed in phosphate-buffered saline at room temperature and blocked for 1 h in 10% serum and 0.03% Triton X-100 in phosphate-buffered saline. Sections were incubated with primary antibody in 2% serum and 0.03% Triton-X in phosphate-buffered saline overnight at 4°C. Primary antibodies to tyrosine hydroxylase (AB152; Millipore; 1:500), NeuN (MAB377; Millipore; 1:200), GFAP (1:400; 13-0300; Invitrogen), and hSNCA (MAB5320; Millipore; 1:400) were incubated at 2% serum and 0.03% Triton-100 in phosphate-buffered saline for 1 h at room temperature. Sections were stained with Alexa Fluor fluorescent secondary antibodies matched to the host primary (Alexa Fluor 488, 555, 568, 633) (1:400; Jackson Immunoresearch, West Grove, PA, USA) in 2% serum and 0.03% Triton-X at room temperature for 1 h. All sections were mounted onto Superfrost Plus slides (Fischer Scientific, Pittsburgh, PA, USA) and cover slipped with Fluoroshield with DAPI (F6057; Sigma). Images were captured on Leica Leitz Digital Module R fluorescent microscope (Leica Microsystems, Buffalo Grove, IL, USA) connected to a Olympus DP72 camera (Olympus, Melville, NY, USA) using the Olympus DP2-BSW software (Olympus). Immunopositive cells were counted per field of view at 40× using ImageJ by two blinded investigators. Cell counts were compared *via t*-tests.

### Semiquantitative PCR

Reverse transcription (High Capacity cDNA Reverse Transcription Kit; Applied Biosystems, Foster City, CA, USA) was performed on total RNA collected from brain regions of interest using a standard stem-loop PCR primer (56) designed to identify miSyn4 or hSNCA. Complementary DNA was subjected to reverse transcriptase-PCR with a standard reverse primer (5′ GTGCAGGGTCCGAGGT) and a forward primer (5′ CACAGATGGGTG ATTGCTTGCTGC) to identify miSyn4 expression compared with β-actin. Random-primer first-strand complementary DNA synthesis was performed using 1 µg of total RNA (High Capacity cDNA Reverse Transcription Kit; Life Technologies) per the manufacturer’s instructions. Assays were performed on a sequence detection system using primers/probe sets specific for mouse SNCA (Mm01188700_m1), hSNCA (Hs002040907_m1), or mouse β-actin (Mm01205674_g1) (ABI Prism 7900 HT and TaqMan 2× Universal Master Mix; Life Technologies).

### Western Blot Analysis

Protein was harvested using radioimmunoprecipitation assay buffer (Pierce, ThermoScientific, Pierce, Rockford, IL, USA) and 1× protease inhibitor using standard techniques and quantified using DC Protein Assay (BioRad Laboratories, Hercules, CA, USA). Protein extracts were separated on a 4–12% Bis-Tris Gel with 2-(*N*-morpholino)ethanesulfonic acid (Invitrogen, Carlsbad, CA, USA) and transferred to Immobilon 0.45-µm polyvinylidenefluoride transfer membranes (Millipore, Bedford, MA, USA). Primary antibodies to hSNCA (1:1,000; S3062; Sigma or 1:2,000; MAB5320; Millipore) and β-actin (1:10,000; A5441; Sigma, St. Louis, MO, USA) were used. Blots were developed using electrochemiluminescence Prime Western Blotting Detection System (GE Healthcare, Buckinghamshire, UK) and quantified by VersaDoc 5000 MP (BioRad Laboratories). All samples were compared *via t*-tests.

### Behavioral Analysis

All behavioral analyses were done at 90 days post-viral injection (AAV2-miSyn4, AAV2-mCherry, or AAV2-eGFP). Behavioral analyses for mice included testing of open-field activity, balance beam testing, and rotarod. Rodents were placed in an open-field arena, and their first 20 min of behavior was captured *via* real time video tracking to prevent habituation and observe activity in novel environment. Balance beam testing was performed as mice traversed a 30″ narrow beam. The time to traverse the beam and the number of falls were scored as failures. Rodents were tested on a rotarod apparatus (Med Associates, St. Albans, VT, USA) with three trials per day as acceleration was uniformly increased from 4 to 40 rpm over 5 min. The trials were stopped at 500 s. Latency to fall (or if mice hung on for two consecutive rotations without running) was recorded for each mouse per trial. Amphetamine based circling was measured 30 min after injected 1 mg/kg of amphetamine IP and by counting the number of circles in 10 min. All behavioral comparisons were performed *via t*-tests or a generalized linear mode.

## Author Contributions

Study design: NN, BD, Y-CK, RB, and AM; data acquisition and analysis: Y-CK, AM, LL, S-WH, and MK; drafting of the manuscript: Y-CK, AM, and NN.

## Conflict of Interest Statement

The authors declare that the research was conducted in the absence of any commercial or financial relationships that could be construed as a potential conflict of interest.
